# Co-designing community-level integral interventions for active ageing: a systematic review from the lens of community-based participatory research

**DOI:** 10.1186/s12889-024-18195-5

**Published:** 2024-03-01

**Authors:** Gubing Wang, Fangyuan Chang, Zhenyu Gu, Dena Kasraian, Pieter J. V. van Wesemael

**Affiliations:** 1 Department of Medical and Clinical Psychology, Tilburg University, Tilburg, Netherlands; 2 Department of Built Environment, Urbanism and Urban Architecture, Eindhoven University of Technology, Eindhoven, Netherlands; 3 School of Design, Shanghai Jiao Tong University, Shanghai, China

**Keywords:** Community-based interventions, Participatory design, CBPR principles, Systematic review, Co-design, Active ageing, Behaviour change, Older adults, Elderly, Design considerations

## Abstract

**Background:**

While community-level interventions for promoting active ageing have received increasing attention and there is a trend to leverage technology to support traditional physical or social interventions, little hands-on guidance exists for designing these integral interventions. This study aimed to examine the interventions reported in the literature guided by Community-Based Participatory Research (CBPR) principles. The goal is to extract insights that inform future practices in co-designing integral interventions for active ageing.

**Methods:**

The systematic review focused on community-level interventions promoting active ageing that integrated physical, social, and digital elements, i.e., integral interventions. Preferred Reporting Items for Systematic Reviews and Meta-Analyses (PRISMA) guidelines were followed. The included interventions were analysed abductively based on the CBPR principles.

**Results:**

A total of 13 studies were included, and 24 design considerations were generated under eight categories. Further reflection identified the interrelated nature of these design considerations and pinpointed the gaps in current research. This study highlights the urgency and importance of sharing recruitment methods and resource allocation details, recording and reporting collaboration specifics, and disseminating findings to stakeholders beyond academia.

**Conclusions:**

This study offers valuable insights and practical guidance to researchers and practitioners developing community-level integral interventions for active ageing. The findings also serve as a starting point for accumulating knowledge and practice in co-designing integral interventions for active ageing at the community level. The next crucial phase involves evaluating these design considerations within real-world cases to assess their applicability and identify potential areas for improvement.

**Supplementary Information:**

The online version contains supplementary material available at 10.1186/s12889-024-18195-5.

## Background

Promoting healthy and active ageing is a public health priority worldwide [1]. In response, community-level interventions have gained increasing attention, working toward establishing an environment that caters to the needs of older adults (OA) and fosters their health and well-being. Community-level interventions (sometimes called community-based interventions) for promoting active ageing refer to the programmes, initiatives, or activities designed to promote health and well-being among OA within a specific geographic area or community [2]. These interventions leverage the strengths and assets of the community, acknowledging OA as valued members of the society deserving opportunities for health, participation, and social inclusion [3]. Through these community-level interventions, OA have access to supportive environments, opportunities for social engagement, and resources that promote their health and quality of life [4]. Moreover, they also help OA in disadvantaged areas age with dignity, remain independent, and contribute meaningfully to society [5]. Furthermore, these interventions could foster intergenerational connections and create age-friendly communities that benefit people of all ages [6].

Most community-level interventions typically focus on social, physical, or digital elements. Social interventions, including discussion groups, volunteer work, and cultural events, provide opportunities for connection and engagement, helping foster relationships between OA and the community [7, 8]. Physical interventions, such as walking groups, exercise classes, yoga sessions, and other group activities, focus on maintaining good physical health, while some OA also find social connections from these activities [9, 10]. Digital interventions, encompassing internet-based communication channels and physical training programs, offer new ways for OA to connect with others from a distance and engage in otherwise inaccessible activities [11].

While supporting active ageing, these interventions can be enhanced by integrating physical, social, and digital elements. For instance, a physical activity program with in-person and online sessions, followed by a social gathering, caters to diverse preferences among OA. This accommodates those inclined towards physical activity, seeking social interactions, preferring face-to-face engagement, or needing remote participation due to travel constraints. The term “integral interventions” is introduced in this study to signify community-level approaches encompassing physical, social, and digital components for promoting active ageing.

Unlike multi-level or multi-component interventions, integral interventions are a type of complex interventions that focus explicitly on the synergy of physical, social, and digital elements to achieve the intended effect. In addition to catering to the heterogeneity of the ageing population [12], an integral intervention is more likely to adapt to the dynamic and complex nature of communities, as they have different resources, community governance structures, sociocultural values, and physical environments [13]. Therefore, developing and evaluating such integrated interventions represents a promising avenue for future research and innovation in active ageing. Despite these interventions’ potential to effectively address OA’s complex needs, little guidance exists on practical frameworks and insights for designing them [14].

Community-Based Participatory Research (CBPR) is a research approach that involves collaboration between researchers and community members throughout the research process and is widely used in public health, urban design and various fields where community engagement is essential for research outcomes [15–17]. We hypothesise that CBPR can offer an insightful perspective in co-designing integral interventions with communities as it recognises the importance of collaboration between researchers and communities to identify and address health and social issues in the community, emphasises that communities are essential units of identity and posits that active community involvement can improve the effectiveness of interventions by building on strengths and resources within the community [18, 19]. While different CBPR principles have been developed, e.g., for healthcare partnerships [20], education [21], and HIV/AIDS partnerships [22], few principles target the promotion of active ageing at the community level. After reviewing the existing CBPR principles, we conclude that the CBPR principles proposed by Israel et al. [23] are the most general and do not focus on a specific group; thus, they can cover our target community. Researchers have widely applied these principles to collaborate with communities to tackle complex health and social challenges [24]. All the principles are shown in Table 1, and each was allocated a keyword for convenient reference in the subsequent text.

**Table 1:** The ten CBPR principles with the keywords in brackets [23]

Principles	
1. Recognises Community as a Unit of Identity (Community)	
2. Builds on Strengths and Resources within the Community (Resources)	
3. Facilitates Collaborative, Equitable Partnership in All Research Phases and Involves an Empowering and Power-Sharing Process That Attends to Social Inequalities (Collaboration)	
4. Promotes Co-learning and Capacity Building among All Partners (Co-learning)	
5. Integrates and Achieves a Balance between Research and Action for the Mutual Benefit of All Partners (Mutual benefits)	
6. Emphasises Public Health Problems of Local Relevance and Ecological Perspectives That Attend to the Multiple Determinants of Health and Disease (Inclusion)	
7. Involves Systems Development through a Cyclical and Iterative Process (Flexibility)	
8. Disseminates Findings and Knowledge Gained to All Partners and Involves All Partners in the Dissemination Process (Dissemination)	
9. Requires a Long-term Process and Commitment to Sustainability (Continuity)	
10. Addresses Issues of Race, Ethnicity, Racism, and Social Class and embrace “cultural humility” (Sensitivity)	

Therefore, in this study, we systematically reviewed studies reporting integral interventions based on the CBPR principles by Israel et al. [23] to inform future practices in co-designing integral interventions for active ageing. We postulate that developing integral interventions promoting active ageing is not only about understanding the specific needs and resources of OA but also about the context and other stakeholders involved in the process. The research question is: What are the design considerations for developing community-level integral interventions for promoting active ageing? The contribution of this study is two-fold. First, it gives an overview of the status quo of integral interventions for active ageing and provides design considerations for developing these interventions. Moreover, a deeper analysis unveiled the interconnected nature of these design considerations and pinpointed gaps within the existing research landscape.

## Methods

### Search strategy and eligibility criteria

A comprehensive search strategy was implemented to minimise publication bias, encompassing multiple databases, grey literature, and conference proceedings. The final searches were conducted in three electronic databases on 19th May 2023: Web of Science, Scopus, and Association for Computing Machinery (ACM) Library. Web of Science and Scopus are known for their comprehensive coverage of peer-reviewed articles. The ACM Library specialises in technological/digital interventions and has access to cutting-edge and peer-reviewed conference proceedings in this field. We developed and tested different search strings to create a search strategy. This strategy included keywords related to ‘community-based initiatives’ and ‘older adults,’ which can be found in Additional File 1. We explored adding a search string with individual terms related to ‘integral interventions,’ but this approach excluded many relevant articles. Therefore, we decided not to include the concept of integral interventions in the search strings but to use it as an inclusion criterion. Similarly, we did not use search terms related to participatory design; instead, the inclusion criterion “the intervention is developed by and with the community” was applied.

In addition to the primary search terms, secondary search strategies were employed, such as forward and backward snowballing, reviewing recent conference proceedings and journals to identify relevant studies, and conducting selective author searches for identified articles’ authors. These additional techniques helped avoid excluding potentially relevant articles and ensure that the literature search was comprehensive. Articles were eligible if they met the inclusion and exclusion criteria in Table 2. Specifically, we excluded studies published more than 6 years ago because significant developments in community-based interventions that integrated physical, social, and digital elements emerged only after 2017. As evidenced by a systematic review of reviews in 2017 [4], internet interventions promoting community-level active ageing were emerging. Focusing on literature published in the last 6 years helps us understand the latest developments in this field. We used 55+ as an age cutoff because, in many contexts, people aged 55 and older are eligible for certain age-related benefits and programmes, making it a practical cutoff for studying populations that can access interventions promoting active ageing. There was no restriction on the study design.

**Table 2:** Inclusion and exclusion criteria for the systematic review

Inclusion criteria	Exclusion criteria
***Participants:***	
Adults aged 55 and older.	Adults aged 55 and older living in institutions.
***Intervention:***	
The intervention is based in a community.	The intervention is not based on a community.
The intervention is developed by or with the community.	The intervention is developed by researchers only.
The intervention consists of physical, social, and digital elements.	The intervention misses physical, social, or digital elements.
***Comparison:*** N/A	N/A
***Outcome:***	
Focus on the development or evaluation of an intervention addressing active ageing.	The intervention was developed for other purposes.
***Others:***	
Original research published in peer-reviewed journals or conference proceedings.	Papers based on secondary data (e.g., reviews) or published in abstracts, posters, books or magazines (i.e., wrong format).
Full-text available.	Full text that is not retrievable.
Written in English.	Papers that use languages other than English.
Published from 19 May 2017 to 19 May 2023.	Journals that are not accessible online.

### Study selection and quality assessment

Two authors (GW and FC) conducted literature searches and imported all references into the Mendeley reference management database. Duplicates, records in other languages, in the wrong formats, or with the wrong target population were then excluded. GW and FC examined titles and abstracts, and to ensure consistency, a pre-test screening was conducted on 80 randomly selected titles and abstracts, resulting in a Kappa k coefficient of 0.8512, indicating “excellent” inter-rater agreement. These two authors then individually assessed the remaining titles and abstracts, and any disagreements were resolved through discussion with the third author (ZG). Studies with no community-level intervention, missing one of the digital, physical, or social elements, whose main outcome is not active ageing, with the wrong target population or in the wrong formats, were excluded. Retrieval was subsequently conducted, after which 6 out of 85 records were excluded because the full texts were absent. GW and FC reviewed the remaining papers independently and discussed whether each paper met the inclusion and exclusion criteria. Uncertainties around paper inclusion and exclusion were again discussed with the ZG until a consensus was reached, leaving a set of 13 papers.

The Mixed Methods Appraisal Tool (MMAT) was used independently by two authors (FC and GW) to assess the quality of the research [25]. This verified checklist is intended for the appraisal of systematic mixed studies reviews. We considered sampling procedures, sample representativeness, and measurement appropriateness when assessing the quantitative descriptive research. We evaluated the applicability of the data sources, analysis method, context-taking approach, and researcher influence for the qualitative studies. The quality of the qualitative and quantitative components was assessed in mixed-methods research. The score ranges from 0 to 7, with a higher score indicating a higher quality. The two authors (FC and GW) and a third author (ZG) had a discussion to settle any last uncertainties or discrepancies over the assessments.

### Data extraction and analysis

Abductive thematic analysis was employed to extract and analyse the data from the selected studies. This method is flexible and adaptable to different research designs, for which an 8-step approach was proposed and followed in this study [26]. Specifically, GW and FC independently extracted data regarding the characteristics of the included studies using a data extraction sheet created using MS Office Excel 2021. The data extraction sheet contains information about the study design, the intervention studied, the participants and how they were involved in developing the intervention. Absent or unclear information was left blank in the table. Discrepancies were resolved through discussions with the third author (ZG). Given the heterogeneity across the reviewed studies, we analysed the studies without further grouping.

GW and FC independently extracted the data linked with the ten CBPR principles and underwent a repeated iterative process to identify the themes and subthemes. The coding schemes from the two independent reviewers were then compared, with any differences in patterns discussed between the reviewers; ZG was consulted, when necessary, until a consensus was reached on the categorisation and definition of each theme and its associated subthemes (i.e., design considerations). Member checking was then used to ensure the validity and reliability of the identified categories [27]. A Sanky diagram was used to visualise the connections of reviewed studies with CBPR principles.

The protocol was developed by GW and FC but was not registered. The review was also not registered. The reporting transparency was checked throughout the review process to ensure that potential biases in reporting were systematically identified and addressed [28]. The completed PRISMA checklist can be found in Additional file 2.

## Results

**Fig. 1:**
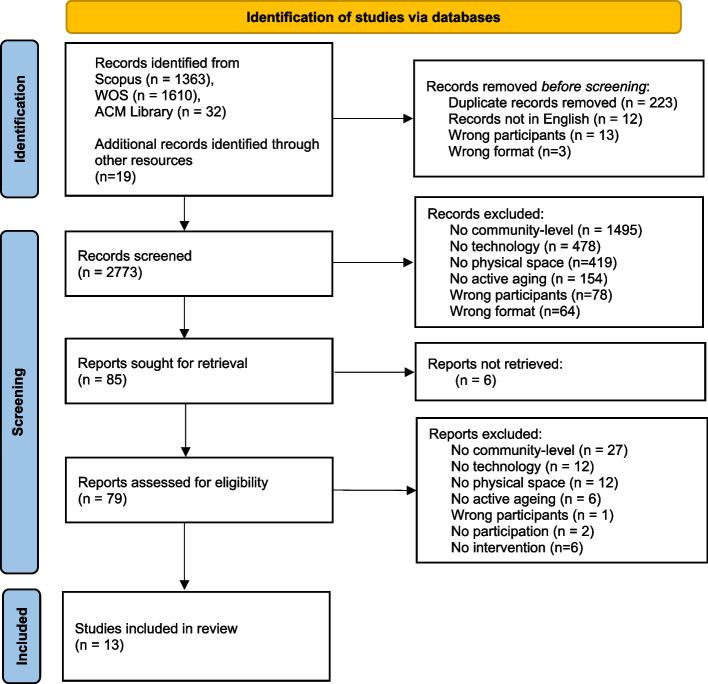
The PRISMA flow diagram

The search yielded 2773 records in the initial screening stage. A further 2688 records against the inclusion criteria were removed through title and abstract screening. Six of the remaining 85 reports were not retrieved, and 66 against the inclusion criteria were excluded, leaving a set of 13 papers (Fig. 1). An overview of the study characteristics is shown in Table 3.

**Table 3:** Study characteristics (for each intervention, the digital elements are boldened, and the physical elements are italicized to distinguish them from the social elements; *OA:* Older adults)

Authors, year	Country	Study Design	Intervention	Participants	Involvement
**Frei et al., 2019** [29]	Switzerland	Action research, implement the intervention and evaluate its effectiveness, feasibility, acceptability, and sustainability	A community walking programme based on the* existing walking routes* and organised with **WhatsApp**	29 OA, local key persons from the local policy and service organisations for OA	Focus group with 4 OA; interviews with eight other key stakeholders; group walking of 13 OA; 6-month follow-up, 11-month follow-up
**Gooch et al., 2021** [30]	United Kingdom	Action research, explore and evaluate the intervention concept	Using **community displays** to communicate and promote physical activity in a *neighbourhood*	25 OA and community workers in four design workshops (each workshop has 5–7 people)	Four workshops with five pre-defined storyboard scenarios
**den Haan et al., 2021** [31]	The Netherlands	Action research, explore and evaluate intervention	A *physical community* learning approach to support OA in using and adopting the **smartphone application GoLivePhone**	7 OA	Living lab approach; a series of focus groups
**Keirnan et al., 2019** [32]	Australia	Action research, explore the intervention concept	A **system** for managing the use of* public space* for social activities in a retirement village	7 OA	Co-design workshops using LEGO serious play methods
**Kosurko et al., 2021** [33]	Canada	Ethnographic research, understand the intervention in real life	A dancing programme in the* neighbourhoods* that allows **online** and offline participation	289 participants (OA, people living with dementia, family carers, admins and staff, volunteers)	Observation, interviews, focus groups, research team reflections
**Lazar et al., 2021** [34]	United States	Ethnographic research, understand the intervention in real life	The creation of a makerspace: a* public place* in the community where people get together to make things (with **technology**)	17 OA	OA involved in deciding what machines to buy after a space was found for this intervention
**Lenstra, 2017** [35]	United States	Ethnographic research, understand the intervention in real life	**Technology** support services in *public libraries and senior centres* that are adapted and reconfigured by OA	209 OA (observation), 54 OA (interviews), and 7 staff (interviews)	OA organised themselves, and no researcher involved in developing the intervention
**Mao et al., 2020** [36]	United Kingdom	Ethnographic research, understand the intervention in real life	A community for sharing, learning and managing music using **digital technology** where people can also meet face-to-face in a *public space*	11 OA (diary-aided interviews)	OA organised themselves, no researcher involved in developing the intervention
**O’Brien et al., 2021** [37]	Ireland	Action research, explore, implement and evaluate the intervention	Community-led walking programme in the *neighbourhood* utilising **activity trackers**	11 OA	Co-design; individual phone interviews following their involvement in the 6-week program, summative evaluation
**Pedell et al., 2021** [38]	Australia	Action research, explore intervention concepts	1) **wearables** for OA to be physically active in the *neighbourhoods*, 2) a social prescribing **system** in the *neighbourhoods*	1) 8 OA 2) 3 OA together with other generations	Interviews and workshops
**Reuter et al., 2019** [39]	United Kingdom, Australia	Action research, explore, evaluate and implement the intervention	**Digital tools** developed to help an OA *community station* run a radio show	8 OA	Observation, interviews, co-design, formative evaluation
**Reuter et al., 2020** [40]	United Kingdom	Ethnographic research, understand the intervention in real life	A *community radio station* where people meet face-to-face, and some use **technology** to digitalise the content	9 OA and 3 radio station managers	Researchers hosted a radio festival during which interviews and observations were conducted
**Yang et al., 2020** [41]	United States	Action research, explore and evaluate the intervention concept	**A digital group game** (Team Bingo) encouraging physical movements in a *senior centre*	9 OA and 2 staff (interviews); 7 OA and 1 staff (evaluation)	Observation, interviews, formative evaluation

### Study characteristics

Table 3 shows six studies were published in 2021, 3 in 2020 and 2019, respectively, and 1 in 2017. All the studies were conducted in Western countries. The study design was either action research or ethnographic research. For action research, an intervention was developed during which evaluation was performed to evaluate the intervention or the concept of the intervention. For ethnographic research, researchers have sought to understand the interventions developed in real life, and these interventions can be developed either by OA or by the collaboration of OA with other partners (e.g., researchers). The contents of the interventions vary across studies. In Table 3, for each intervention, the digital elements are highlighted in bold text and the physical elements in italics text to distinguish them from social elements and generate an overview of the integral interventions’ physical, social, and digital elements. The number of OA involved was approximately 10–30 for most of the studies, except for two ethnographic studies involving more than 200 OA [33, 35]. The participants were usually involved in interviews, focus groups, co-design workshops and observations during the intervention development. Only two studies mentioned the setup of co-design workshops [30, 32].

### Quality assessment

In general, the studies vary in quality, with some demonstrating strong adherence to methodological standards while others exhibit limitations in certain aspects of study design and reporting. All studies satisfied the screening questions. The criteria differ per study approach (i.e., qualitative, quantitative and mixed methods). As shown in Table 4, four studies have a score of 5, four have a score of 6, and five have a score of 7, respectively. Since the evaluation criteria differ depending on the study approach, the approach of each study is presented in Table 4, and their corresponding criterion numbers in the checklist are shown in the last column.

**Table 4:** Overview of study quality scores (Y: meet the requirements, N: do not meet the requirements)

Authors, year	Approach	Score	Criterion number for the corresponding study approach (Y/N)
**Lenstra et al., 2017** [35]	Qualitative	5	1.1 (Y) 1.2 (N) 1.3 (Y) 1.4 (Y) 1.5 (N)
**Pedell et al., 2021** [38]	Qualitative	5	1.1 (Y) 1.2 (N) 1.3 (N) 1,4 (Y) 1.5 (Y)
**O’Brien et al., 2021** [37]	Qualitative	5	1.1 (Y) 1.2 (N) 1.3 (N) 1,4 (Y) 1.5 (Y)
**den Haan et al., 2021** [31]	Qualitative	5	1.1 (Y) 1.2 (Y) 1.3 (N) 1,4 (Y) 1.5 (N)
**Reuter et al., 2019** [39]	Qualitative	6	1.1 (Y) 1.2 (N) 1.3 (Y) 1,4 (Y) 1.5 (Y)
**Yang et al., 2020** [41]	Mixed methods	6	5.1 (Y) 5.2 (Y) 5.3 (Y) 5.4 (N) 5.5 (Y)
**Keirnan et al., 2019** [32]	Qualitative	6	1.1 (Y) 1.2 (Y) 1.3 (N) 1.4 (Y) 1.5 (Y)
**Gooch et al., 2021** [30]	Qualitative	7	1.1 (Y) 1.2 (Y) 1.3 (Y) 1.4 (Y) 1.5 (Y)
**Frei et al., 2019** [29]	Mixed methods	7	5.1 (Y) 5.2 (Y) 5.3 (Y) 5.4 (Y) 5.5 (Y)
**Mao et al., 2020** [36]	Qualitative	7	1.1 (Y) 1.2 (Y) 1.3 (Y) 1.4 (Y) 1.5 (Y)
**Kosurko et al., 2022** [33]	Qualitative	7	1.1 (Y) 1.2 (Y) 1.3 (Y) 1.4 (Y) 1.5 (Y)
**Reuter et al., 2020** [40]	Qualitative	7	1.1 (Y) 1.2 (Y) 1.3 (Y) 1.4 (Y) 1.5 (Y)
**Lazar et al., 2021** [34]	Qualitative	7	1.1 (Y) 1.2 (Y) 1.3 (Y) 1.4 (Y) 1.5 (Y)

### Main findings

**Fig. 2:**
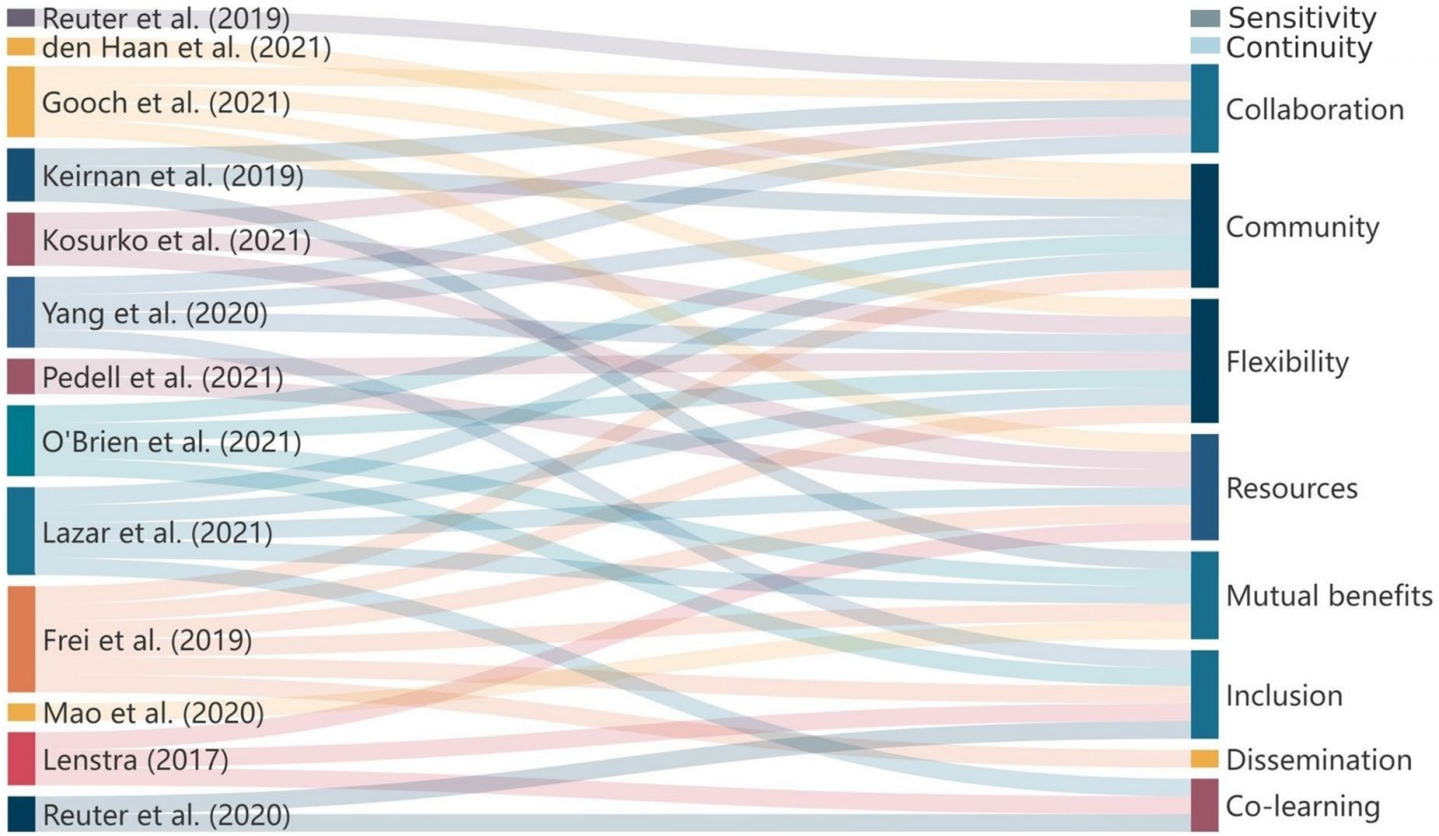
The connections between the reviewed studies and CBPR principles

The reviewed studies connected with the CBPR principles to various extents, while no studies covered or reported on the last two CBPR principles, namely, “Requires a Long-term Process and Commitment to Sustainability (Continuity)” and “addresses Issues of Race, Ethnicity, Racism, and Social Class and embrace cultural humility (Sensitivity)” (Fig. 2). Twenty-four design considerations were generated in eight categories (themes) based on how the eight CBPR principles were followed, as reported by the reviewed studies (Table 5). Details of the categories, design considerations and associated example evidence from the reviewed studies can be found in Additional File 3. The definition of each category is slightly different from that of each CBPR principle because the design considerations are for co-designing integral interventions rather than for CBPR. Each subsection below will elaborate on one category of design considerations.

**Table 5:** Design considerations when developing community-based integral interventions

Categories	Design considerations	Studies
**Community: if the intervention recognised the community as a unit of identity ​**	Recruit participants^a^ via the commonly used channels that are specific to the target community	[29, 31, 41]
	Understand the values and norms of the community	[29, 30, 32, 34–36, 41]
	Enable connections among stakeholders^b^ of the community	[29, 30, 32, 41]
**Resources: if the intervention was making use of the resources that the community already have**	Utilise physical resources of the community	[29, 30, 35]
	Utilise social resources of the community	[30, 31, 33, 34]
	Understand the history and politics of resource allocation within the community	[32, 34, 35, 38]
**Collaboration: if all stakeholders worked together to develop this intervention throughout**	Co-design the intervention with key stakeholders	[29, 30, 33, 38, 41]
	Match the level of co-design based on the shared goals of stakeholders	[29, 30, 34, 37–39, 41]
	Help stakeholders to envision future scenarios	[30, 32, 41]
**Mutual benefits: if all stakeholders gained benefits from the intervention**	Help participants concretise the benefits of the intervention in their daily lives	[29, 36, 37]
	Uncover reasons behind tension among stakeholders (if any)	[32, 34]
	Clarify whose interests the stakeholders represent	[30, 34]
**Co-learning: if the intervention provided suitable education and training and allowed knowledge exchange**	Scaffold education and training based on the current knowledge, preferences, and experiences of participants	[34, 37]
	Cultivate a collaborative learning culture in the community	[35, 36, 40]
	Facilitate learning between communities with similar interests	[34, 40]
**Flexibility: if the intervention was developed gradually with feedback cycles​**	Start small and set milestones to track the progress	[29, 30, 33]
	Collect feedback and ideas from key stakeholders regularly and iterate the intervention when needed	[29, 34, 38, 41]
	Understand the reasons for dropping out	[29, 37]
**Inclusion: if the intervention has considered a wide range of physical, cognitive, socio-economical, cultural factors and personalities of participants**	Uncover and tailor to the diversity of participants	[29, 30, 37]
	Recognise any ageism views held by the stakeholders and researchers themselves	[35, 40]
	Know what technologies the participants are familiar with (have both technical and non-technical options for participants if needed)	[34, 40]
**Dissemination: if the intervention and knowledge gained are communicated to all stakeholders**	Provide periodical findings to stakeholders	[29]
	Create a practical guide to help communities implement the intervention	[29]
	Train the responsible person(s) for intervention handover in sufficient time	[29]

^a^With participants, we mean people who will participate in the intervention; in our case, they are older adults

^b^With stakeholders, we mean participants and individuals involved in organising, maintaining, and creating conducive conditions for the intervention

### Community

In the context of established communities, researchers need to recognise and respect each community’s unique identity. The first design consideration is to recruit participants via the commonly used channels specific to the target community. It has been found that each community has its channels for information dissemination and communication. For example, Yang & John (2020) approached a senior centre to recruit participants [41], and den Haan et al. (2021) recruited participants via a local association and local newspaper [31]. Frei et al. (2019) tried diverse channels, including local events for OA, local newspapers, flyers to the mailboxes of OA, and a pharmacy poster [29]. Community members usually stay up-to-date and trust the information disseminated from these channels, which can lead to higher levels of interest and participation.

The second design consideration is to understand the values and norms of the community. Shared values and norms play an important role in the psychological sense of community [42]. Most studies conducted observations, interviews or focus groups to understand what the community members think is important in life and what they regard as usual [29, 30, 32, 34–36, 41]. For example, researchers learned from their observations and interviews that “social connectedness” and “physical health” are vital to OA in the senior centre, and Bingo is the most popular game they play. Hence, they developed an intervention called Team Bingo, which aims to “reduce social isolation and increase physical activity” for OA in a community setting [41].

The third design consideration is to enable connections among stakeholders of the community. Many studies have explored different ways to connect key stakeholders of the community in their interventions. For instance, Frei et al. (2019) established a WhatsApp group to facilitate communication among participants and organisers, where scheduling walking activities emerged as a popular topic [29]. Gooch et al. (2021) aimed to enhance participants’ sense of connectedness through a community display [30]. Keirnan et al. (2019) organised a co-design workshop leading to intervention concepts for improving communication between OA and community managers [32]. O’Brien et al. (2021) highlighted that their intervention, focused on tracking physical activities, received feedback from participants emphasising the importance of social connections for program enjoyment and engagement [37]. Lazar et al. (2021) reported that even though OA were invited to co-design a makerspace, many OA reported that they did not want a makerspace in the first place; they missed communication with managers regarding their needs [34].

### Resources

This category focuses on how to make use of the resources of the community during intervention development. The first design consideration is to utilise the physical resources of the community, i.e., resources in the community’s physical environment. For example, Frei et al. (2019) used existing walking trails in the neighbourhood [29]. Gooch et al. (2021) conducted workshops at a location where community members usually meet [30]. Lenstra (2017) found that OA socialised more in senior centres than in public libraries when they learned technology and received support on technology use [35], which indicates how the physical environment can shape the social atmosphere.

In parallel to the first design consideration, the second design consideration is to utilise the social resources of the community. We define community social resources as the value derived from member relationships, shared knowledge, exchanged skills, and members’ potential ability to obtain resources, favours, or information from their personal connections. For example, Lazar et al. (2021) observed that during the formation of the makerspace, community members with technological expertise were selected for the committee, and beyond the committee, some members supported this initiative through donations and sharing their expertise [34]. Gooch et al. (2021) encouraged OA to organise walks and recruit new members via their connections [30]. In the smartphone learning program, OA previously trained in using technology offered classes to other OA in the community [31]. In the Sharing Dance program, facilitators were identified locally for each site [33].

The third design consideration is understanding the community’s history and politics of resource allocation. Understanding how resources have been allocated in the past and who makes or influences decisions is important for successful resource utilisation. For instance, Pedell et al. (2021) explored resources, enablers, and barriers to social prescribing services in the community [38]. Lenstra (2017) highlighted ageist policy in public libraries, where volunteers for technical support are predominantly recruited from universities, mainly in their 20s [35]. This practice perpetuates the notion that OA should seek help from younger generations rather than peers for technology assistance. To counteract ageist policies, OA organised themselves in a community to pressure the local government for financial support to form new senior centres for technology learning. To understand local politics, some researchers have also investigated how decision-makers are perceived by community members [32, 34].

### Collaboration

This category ensures that key stakeholders work together to develop the intervention. The first design consideration is to co-design the intervention with key stakeholders. Involving key stakeholders actively and early in the development process can generate creative ideas, facilitate consensus, and promote collaboration. Many studies worked with OA collaboratively, and many of them involved other stakeholders during co-design, such as community workers [30], staff in the senior centre [41], staff in social prescribing services [38], and key persons from local policy and service organisations [29]. Kosurko et al. (2022) involved community administrators, family carers, volunteers, and staff facilitators in addition to OA [33].

The second design consideration is to match the level of co-design based on the shared goals of stakeholders. Three studies started the co-design based on a concept that researchers had already drafted, for example, walking activity [29], social prescribing [38], and community displays [30]. The stakeholders were involved in co-design the details of these concepts; three studies started without preconceived concepts from the researchers, used co-design to understand the challenges of the community, and interventions were then developed [38, 39, 41]; one study helped the community to realise its initiative, where the researchers positioned themselves as facilitators [37]. As illustrated by the studies above, three levels of co-design emerged in our review. When the level of co-design did not match the shared goals, resistance and scepticism from under-involved groups might occur [34].

The third design consideration is to help stakeholders envision future scenarios. Gooch et al. (2021) used storyboards to help OA and community workers understand how a community display could encourage physical activities [30], and participants were given templates to create storyboards by themselves. Keirnan et al. (2019) used LEGO to encourage residents of the retirement park to build an ideal park in their minds, based on which the researchers identified the residents’ wishes and the current challenges [32]. Yang & John (2020) developed a prototype of their intervention and presented it to OA and staff at the senior centre for a test play to help them understand what roles the intervention can play in the future [41].

### Mutual benefits

This category focuses on ensuring that all stakeholders gain benefits from the intervention. The first design consideration is to help participants concretise the benefits of the intervention in their daily lives. This is the most common for studies focusing on promoting physical activities, as some regard exercise as pain, and it is hard for some participants to keep envisioning the long-term gain of physical exercise. O’Brien et al. (2021) meet participants regularly to explain the benefits of physical activities in their daily lives [37]. Frei et al. (2019) invited each OA to set short- and long-term goals they wanted to achieve by participating in the intervention [29]. Mao et al. (2020) found that letting OA realise their accumulated competencies by participating in the intervention over time has positive consequences for their self-efficacy and confidence in realising their goals [36].

The second design consideration is to uncover reasons behind tension among stakeholders, if any. Understanding the reasons behind this makes it easier to resolve the conflict by finding common ground; communicating the interests explicitly could also help stakeholders understand each other. For instance, Keirnan et al. (2019) organised a codesign workshop to let residents of a retirement park express their tensioned relationships with community managers using arts and crafts materials [32]. The residents later reported that they had more understanding about the managers, “it is not them who are problematic, it is the system that they are in”. The study on the formation of the makerspace discovered that different stakeholders have varied opinions on what was considered a suitable return on this investment, which partly led to resistance to the intervention [34].

The third design consideration is to clarify whose interests the stakeholders represent. When talking about “OA”, participants might think of people who are older than them, and they then “design for others” rather than “design for themselves”. It is also valuable when people design for others, yet it is important to clarify if this is the case. For example, when briefing the community workers during the development of the community display, they were prompted to discuss “what they felt would be useful for the OA they support” rather than how they would support the OA with the community display [30]. The same happens when talking about “community” As Lazar et al. (2021) put it, “There is a complexity to whom participants believed constitutes this community” [34]. Clarifying questions can help researchers understand who is speaking for whom and determine if these imagined others exist and if their interests were predicted correctly.

### Co-learning

This category intends to help researchers provide education and training that empowers participants in the intervention while encouraging knowledge exchange. The first design consideration is to scaffold education and training based on participants’ current knowledge, preferences, and experiences. Especially when participants need to learn the technology in the intervention. For instance, in the intervention using trackers to promote physical activities, the researchers inquired about the previous experiences of participants with tracking devices and then provided tailored support for each participant [37]. While developing the makerspace, it was observed that OA got stuck or gave suggestions unsuitable for a makerspace, i.e., some OA do not know what a makerspace is [34]. This indicates that more scaffolding is needed at the beginning of the co-design sessions. Regarding learning preferences, some OA think learning something new is exciting, while others only learn if they can benefit loved ones or others [34].

The second design consideration is cultivating a collaborative learning culture in the community, i.e., community members can provide education and training to each other. The study on community music found that enabling competence sharing could support the social participation of OA [36]. OA in the community radio intervention also expressed that they enjoy sharing and learning experiences with and from each other [40]. Lenstra (2017) noted that newcomers to senior centres have to be aware that they are expected to participate in technology support services when becoming part of the group, and OA only felt comfortable helping each other when an officially designated “technology helper” was in the room, whom they could turn to if obstacles arose. Therefore, specific criteria need to be met for this collaborative learning culture to develop.

The third design consideration is to facilitate learning between communities with similar interests. For instance, Reuter & Liddle (2020) hosted a festival to connect groups of OA who create radio content with each other [40]. This interconnectedness allows communities to learn from one another, leading to valuable cross-pollination of knowledge and experiences. When establishing the makerspace, Lazar et al. (2021) showcased various makerspace examples from other communities to inspire and engage the participants [34]. By exposing the community members to successful models from elsewhere, the project leader sought to generate interest and ignite creativity among participants. Thus, existing examples from other communities provide valuable lessons and ideas that can be adapted and applied to the specific context of the targeted community.

### Flexibility

This category aims to support researchers in developing interventions gradually with feedback cycles. The first design consideration is to start small and set milestones to track the progress of the development. “Starting small” implies a lower complexity of the intervention and a smaller number of participants. For instance, Gooch et al. (2021) conducted a pilot study to evaluate the storyboards they created, which were revised and ready for the workshop [30]. The workshop collected feedback from stakeholders about the concept before it was realised into a working prototype. Kosurko et al. (2022) conducted six pilot studies of the Shared Dance Older Adult program, and the feedback from each pilot resulted in the evolution of a multimodal program [33]. Milestones can help researchers and stakeholders track and align on the project’s progress. The study of Frei et al. (2019) serves as a good example: intervention design, goal setting with participants, monthly reflection meetings, responsibility transfer, researcher departure, and follow-up [29].

The second design consideration is to collect feedback and ideas regularly from key stakeholders and iterate the intervention when needed. Being regular helps to build a routine and manage the expectations of key stakeholders. For example, Frei et al. (2019) set a monthly reflection meeting during intervention deployment [29]. When designing the social prescribing service, Pedell et al. (2021) organised three workshops for the insights to cascade into the next to ensure that the co-design process was “open and flexible” [38]. Lazar et al. (2021) observed that the committee met monthly to decide on many aspects of the makerspace [34]. Yang & John (2020) involved both staff and OA from the senior centre to ensure that the design suits the needs of both groups [41].

The third design consideration is to understand the reasons for dropping out. Two studies conducted exit interviews to collect this information, which could reveal useful feedback for intervention improvement. For instance, Frei et al. (2019) understood from their exit interviews that the participants dropped out because of adverse events unrelated to the intervention [29], while O’Brien et al. (2021) learned from their exit interviews that participants dropped out due to either the usability of the tracking device or lack of motivation towards the intervention [37].

### Inclusion

This category intends to help researchers ensure that the intervention has considered a wide range of physical, cognitive, socioeconomic, and cultural factors and participants’ personalities. The first design consideration is to uncover and tailor to the diversity of participants. For example, Frei et al. (2019) grouped OA based on their physical capability levels and allocated walking trails with appropriate intensity levels to each group [29]. O’Brien et al. (2021) identified a few factors influencing whether tracking devices can promote physical activities for OA: self-awareness, previous engagement with self-tracking, and whether one is more intrinsically or extrinsically motivated [37]. It has also been found that some OA prefer competition [37], while others prefer collaboration [30] when engaging in physical activities.

The second design consideration is to recognise any ageism views the stakeholders and researchers hold. Sometimes, OA can perceive these ageism views, while other times, they also have ageism views about themselves. The creation of radio stations, according to some OA, is an act that challenges ageism because it is an effective medium to democratise the voices of OA [40]. Meanwhile, they advocated for an intergenerational network, considered intergenerational content choices to connect OA with society, and let society know that OA is not a homogenous group. Lenstra (2017) observed that ageism views appear in policies, institutional structures, and practices, which are usually created by some stakeholders [35]. They found that, among OA, some exert their agency to work around these policies and practices or shape new ones, while others were surprised when they felt that society wanted them at their age. It is important for researchers to be aware of ageist thoughts when stakeholders are not.

Regarding involving technology in the intervention, it is key to know what technologies OA are familiar with and, if needed, include non-technical options as part of the intervention. For instance, Yang & John (2020) only deployed a TV display as the technological element of the Team Bingo game as this is a technology that OA are familiar with [41]. When selecting self-threading machines for the makerspace, some OA stressed that they come to the makerspace to make things rather than to learn technologies; in response, the committee decided to purchase two machines with one set at the default setting so that OA can use it without interacting with the “technical bits” [34]. Reuter & Liddle (2020) identified that radio content creation engages both non-digital (e.g., presenting) and digital (e.g., editing) skills, with broadcasts as a bridge to the digital divide [40]. OA can start with what they are good at and work towards the same goal. The non-digital and human interactions during content creation also provide a safe and accessible environment for OA to learn and practice digital skills. They observed that all members began to engage digitally to stay involved with content creation during the COVID-19 pandemic.

### Dissemination

This category aims to support researchers in communicating the intervention and knowledge gained to key stakeholders. Only Frei et al. (2019) reported the dissemination approach, yet we extracted three insightful design considerations from this study [29]. The first design consideration is to provide periodic findings to stakeholders during intervention development. This differs from and complements the design consideration of “regularly collecting feedback and ideas from key stakeholders”. These two design considerations foster two-way communication between the research team and the community. In addition to presenting the intervention development progress, the researchers can reflect on the procedural knowledge with the community, for example, how the research team and the community can collaborate better.

The second design consideration is to create a practical guide to help communities implement the intervention. After evaluating the feasibility and acceptability of the intervention via interviews, Frei et al. (2019) developed a manual for intervention implementation, which can be disseminated to communities with similar goals of promoting physical activity. This action scales the social impact of their research findings.

The third design consideration is to train the responsible person(s) for intervention handover in sufficient time, as knowledge can be explicit and implicit. Implicit knowledge is hard to express in words but can be transferred by training, such as observing others performing a task, and early involvement helps with knowledge transfer [43]. Frei et al. (2019) started the transfer 5 months before the end of the research, and they trained the responsible persons from organisation skills, technical know-how, and recruitment strategies to fund application techniques.

Regarding reporting biases, the reviewed studies reported how they co-designed integral interventions for active ageing in various detail. Some useful details of co-designing might not be reported, and not all reported details contribute positively to the co-design of these interventions. This limitation will be discussed further in the next section.

## Discussion

Our review identified 13 relevant studies, which connect to 8 of the 10 CBPR principles. Eight categories of 24 design considerations were synthesised based on the included studies and the CBPR principles they connect to.

### Synthesis with existing knowledge

Some of these findings relate to the broader literature. First, our study highlights that recognising the diversity among OA is key, which confirms prior suggestions in the participatory design literature [44, 45]. Our review adds that in addition to tailoring interventions for OA based on their interests and skills, empowering them to adapt the intervention to their competencies and needs is beneficial for inclusivity. Incorporating both approaches can bring the intervention closer to being truly inclusive. Regarding the first approach, in addition to fitness level and digital literacy [46], another dimension of tailoring could be the preferred style of social interaction, as some OA prefer collaboration [30], while others prefer competition [41]. Besides, our findings align with previous research that OA who are more mobile, outgoing and confident tend to participate in co-design workshops and meetings [47]. Therefore, we encourage researchers to employ a wide range of involvement methods to ensure that the voices of more OA in the community are heard. This study also found that building confidence and demonstrating sensitivity to the experiences of OA are vital, which has been put forward in the participation design literature [48].

Moreover, we found that understanding stakeholders’ genuine needs and ensuring that these needs are adequately addressed in the intervention is important. This finding concurs with earlier suggestions by Righi et al. (2018), who discovered that OA may participate in a study not necessarily to benefit themselves but to assist researchers in data collection or other altruistic reasons [49]. In such cases, they may not intend to be potential intervention users.

Furthermore, our findings corroborate the argument that people can creatively deal with the everyday challenges they encounter as they age [50]. Brandt et al. (2010) proposed the concept of “situated elderliness” as a more helpful mindset than viewing “all OA as the same” or “each older adult as different”. “Situated elderliness” acknowledges that as people age, they may encounter difficulties in some activities while still being able to handle others in their daily lives. This might increase OA’s need to belong to a community where “seniors are skilfully enacting everyday practices as seniors” [51]. We add that it is also valuable to involve key stakeholders in communities that are not necessarily OA, such as community workers, senior centre volunteers, and local policymakers, to ensure a comprehensive approach when developing community-level integral interventions for active ageing.

### Aspects for future research

However, our review also revealed some gaps in current research. First, only a few studies reported how participants were recruited, and many researchers identified small sample sizes as a limitation in their studies. For instance, despite employing various channels, Frei et al. (2019) reported achieving less than half of their targeted sample size [29]. To enhance the participation rate, researchers should reflect critically: what other strategies could be employed to improve recruitment? How can the sample size be determined to be representative of the community? Who should researchers consult while devising recruitment plans? The most recent literature on this topic dates back to 2009 [52]. It is crucial for researchers to share detailed descriptions of their recruitment methods, the subsequent outcomes, and their reflections when disseminating their work.

Besides, only a few studies described the resources and their allocations in the community. This information is crucial for future researchers to contextualise the findings of these studies. By assessing the similarities between the communities reported and their target communities, future researchers can potentially adopt relevant research methods and design features from previous research. In this way, the transferability of knowledge in this field could be enhanced. A review of community participation in health planning also highlighted the politics behind resource allocation, namely, decisions are made at many levels (e.g., starting from the national level), and these decisions can be influenced to change resource allocation [53].

Moreover, reviewed studies reported their collaboration with the community in varying detail. Some studies briefly mentioned the participants involved and the number of meetings, while others elaborated on what questions were asked to which stakeholders and what facilitation tools were provided. Similarly, a recent rapid overview of reviews of research co-design in health concluded that co-design has been widely used but seldom described or evaluated in detail [54]. We encourage researchers to record and report these details or attach these details in the appendix so that more insights can be shared on the intricacies of collaborative processes, ultimately fostering more effective collaboration practices.

Furthermore, only one reviewed study reported on research dissemination. A review of the link between research and practice in social work calls for urgently establishing the effectiveness of knowledge utilisation models in various contexts [55]. Disseminating research outcomes beyond academic circles can empower communities to implement interventions and develop future interventions by themselves. Ideally, representatives of each stakeholder group should be involved in co-creating the dissemination materials to ensure their accessibility. Although such activities are pivotal for ensuring the intervention’s long-term impact and sustainability, these may not be feasible in projects with limited time and resources.

The omission of the last two CBPR principles in the reviewed studies has several potential reasons. First, not all proposed principles are universally applicable in every setting and community, as highlighted by Israel et al. [23]. The penultimate principle, “continuity”, emphasises the need for ongoing partnerships beyond a single research project, extending before and after funding. Some reviewed studies could have followed this principle, yet it was not reported. None of the reviewed studies reported the procedural knowledge, i.e., how they maintain the partnership, in detail. We encourage future researchers to reflect and report on this principle. The reason why the last principle, “sensitivity”, was not covered could be that the OA communities involved in the reviewed studies are not the minority in terms of race, ethnicity, racism, or social class. It could also be that this aspect was overlooked. We invite future researchers to consider whether these factors are relevant depending on the type of OA communities they collaborate with.

Overall, the design considerations identified in this study were interrelated in various ways. For example, “co-design the intervention with key stakeholders” could be an effective approach to “uncover reasons behind tension among stakeholders”, and the underlying reasons sometimes are about “the history and politics of resource allocation within the community”. Likewise, the categories created were interrelated as well. For example, “collaboration” is a key approach for ensuring “mutual benefits”; that is, when all stakeholders work together to develop this intervention, it is more likely for the intervention to benefit all stakeholders. By acknowledging these interconnections in a real-world context, all design considerations provide a holistic guide throughout the design process, and there is no sequential order for using these design considerations.

Lastly, many design considerations identified in this paper align with the design strategies proposed previously for long-term collaboration with OA communities [56]. Specifically, the strategies “begin with small but relevant”, “access design”, “build scaffolds”, “build and release prototypes iteratively, rapidly and from early on”, “stay attentive to partial failures and what can be learned from them”, and “avoid design locking-in with crucial choices”. We position our design considerations to complement these strategies and recognise that design is “only one line of development” that can help researchers and communities reach their goals. Researchers should also attend to the other changes in the community during the project span for effective collaboration. We view the developed intervention as the tangible outcome of the collaboration, and the intangible outcome of the collaboration could be a lasting collaboration relationship, empowerment gained by the community, and more harmony within the community.

### Limitations

Our study has a few limitations. Regarding the search results, since the intervention types and user groups varied widely among the reviewed studies, it is difficult to synthesise meaningful comparisons between studies. As the quality of the included studies varies largely, we cannot conclude the effectiveness of these interventions on active ageing. Regarding reporting biases, we acknowledge that the reviewed studies focus on developing interventions rather than evaluating the best ways to develop interventions. By extracting the common practices from the reviewed studies, we form a list of design considerations to be evaluated in practice to gain more knowledge on how to co-design integral interventions for active ageing.

Methodologically, since we limited the language of the search to English, all reviewed studies were found to be conducted in Europe, Australia, or North America; hence, the search results might be biased toward a Westernised view of developing community-level integral interventions. Moreover, we only included three databases in this review due to the time limit. Including more databases, such as PubMed, might lead to more findings. We limited the search to the last 6 years as the most integral interventions for active ageing have been developed since 2017, while a longer period could help with understanding the initial development of integral interventions. Furthermore, we set 55+ as the age cutoff, and we are aware that the definition of OA differs per culture, with some reviews including participants that are 40+ [4]. As many researchers started to advocate not using age as the standard for classifying OA [44], future reviews could relax the inclusion criterion on age and include studies so long as they investigate interventions for active ageing. In addition, our choice of using the CBPR principles by Israel et al. (2018) is shaped by our previous experience and knowledge in working with OA communities, which could have introduced inherent biases. Lastly, the transparency of this study could be improved by registering a protocol beforehand, which could reduce the risk of reporting bias, too. We position this study as a starting point, encouraging future researchers to expand the search scope, e.g., explore other databases and to understand the development of this research field in non-English speaking cultures with corresponding culturally appropriate frameworks.

## Conclusions

To conclude, this study offers valuable insights and practical guidance for co-designing community-level integral interventions for active ageing through the lens of CBPR principles. The findings serve as a starting point for accumulating knowledge and practice in co-designing integral interventions at the community level that enhance the well-being and quality of life of OA. Moreover, our findings highlight the gaps in current research: reporting recruitment methods and resource allocation details, recording collaboration specifics, and disseminating findings beyond academia. To address these gaps, future research should focus on conducting case studies or pilot projects to evaluate these design considerations in diverse community settings.

### Supplementary Information

**Supplementary Material 1.**

**Supplementary Material 2.**

**Supplementary Material 3.**

## Data Availability

The datasets used and analysed during the current study are available from the corresponding author upon reasonable request.

## Abbreviations

CBPR: Community-based participatory research
OA: Older adults
