# Supplementary material for: Co-designing community-level integral interventions for active ageing: a systematic review from the lens of community-based participatory research
Source: BMC Public Health. 2024 Mar 1;24:649. doi: 10.1186/s12889-024-18195-5 (PMC10905784; doi:10.1186/s12889-024-18195-5)
Supplement: Supplementary file 1 — Supplementary Material 1. [file 12889_2024_18195_MOESM1_ESM.docx]

**Additional file 1: Searches in three electronic databases (19 May 2023)**

**WOS (n=1610) [https://www.webofscience.com]**

(communit* OR neighborhood* OR neighbourhood OR "public space" OR "physical environment*" OR "natural environment*")

AND

("older adults" OR elderly OR seniors OR "older people")

AND

(Technolog* OR Digital*)

**Scopus (n=1363) [https://www.scopus.com/home.uri]**

(communit* OR neighborhood* OR neighbourhood OR "public space" OR "physical environment*" OR "natural environment*")

AND

("older adults" OR elderly OR seniors OR "older people")

AND

(Technolog* OR Digital*)

**ACM Library (n=32) [https://dl.acm.org/]**

(communit* OR neighborhood* OR neighbourhood OR "public space" OR "physical environment*" OR "natural environment*")

AND

("older adults" OR elderly OR seniors OR "older people”)

*Note: no search terms related to technology is used for the ACM Library, because this database contains all studies related to technology by default.*
